# Contract teaching as a liminal bridge: how pre-entry beliefs become commitment in PE teacher socialisation

**DOI:** 10.3389/fspor.2025.1719826

**Published:** 2025-12-18

**Authors:** Heng Yeow Yap, Harrie Desianto Hussien, Steven Kwang San Tan, Jernice Sing Yee Tan, Shern Meng Tan

**Affiliations:** Physical Education and Sports Science Department, National Institute of Education, Nanyang Technological University, Singapore Singapore

**Keywords:** occupational socialization, contract teaching, teacher identity, mentoring, teacher retention

## Abstract

**Introduction:**

High rates of teacher turnover continue to pose a significant challenge to the sustainability of physical education (PE) worldwide. This study explored how pre-entry and induction experiences influence preservice PE teachers’ commitment to the profession, drawing on occupational socialisation theory.

**Methods:**

A a convergent parallel mixed-methods design was employed involving 79 preservice PE teachers in Singapore (Postgraduate Diploma, *n* = 48; Diploma, *n* = 31). Quantitative data from self-report measures of Initial Socialised Beliefs (ISB), Contract Teaching Stint Experience (CTSE), and Commitment to Teach (CTT) were analysed using mediation models. Pre-entry experiences were measured using the Initial Socialised Beliefs (ISB) scale (10 items), induction experiences were captured using the CTS Experience (CTSE) scale (5 items), and Commitment to Teach (CTT) was assessed using a 5-item intention scale. Qualitative responses to open-ended items provided explanatory depth and triangulation.

**Results:**

ISB was positively associated with CTT, and CTSE partially mediated this relationship. Teachers who described supportive mentorship, progressive teaching responsibilities, and early classroom success reported higher commitment, whereas administrative overload and weak induction reduced motivation.

**Discussion:**

The findings suggest that structured, high-quality CTSE can transform positive pre-entry beliefs into a lasting professional commitment. Schools and training programmes can strengthen retention by fostering meaningful early-career support through mentor pairing, workload design, and opportunities for authentic teaching success.

**Conclusion:**

The transition from pre-entry to induction represents a pivotal phase in shaping the professional trajectories of PE teachers. Intentional design of school-based experiences—centred on mentorship, workload balance, and feedback—can strengthen teacher retention and sustain the quality of the PE workforce.

## 1 Introduction

High rates of teacher turnover threaten educational stability and student learning worldwide, making the formation of strong early professional commitment a critical concern. A recent study found that pre-service teachers' prior beliefs can shape classroom judgements, underscoring the need to address orientations during initial preparation (1). In physical education (PE), occupational socialisation theory (OST) has offered a widely used framework for explaining how recruits develop through three interconnected phases: acculturation (pre-entry experiences and beliefs), professional socialisation (initial teacher education), and organisational socialisation (induction and workplace learning) (2–5). Acculturation exerts enduring influence, as years of the “apprenticeship of observation” often crystallise common-sense beliefs about the purpose of PE and the identity of the PE teacher. A pressing question, however, concerns how and under what conditions these beliefs gain or lose traction once recruits begin working in real classrooms and departments.

The bridge between acculturation and professional socialisation is comparatively underspecified in empirical research (6). Early, realistic field experiences are often theorised as the crucible where idealised beliefs encounter school politics, appraisal logics, and workload demands. Across international contexts, induction research has predominantly focused on teachers who have already begun full-time work, leaving the formative pre-entry phase largely unexamined. Yet OST suggests this is precisely the period when beliefs and identity are most subject to recalibration, as novices first encounter real school routines, workload expectations, and mentoring logics. Despite the growth of early-immersion pathways and residencies, few studies test this transitional space using designs that integrate both statistical evidence and participants' explanatory narratives. Little is known about how these encounters transform initial beliefs into (or away from) professional commitment, leaving a major conceptual and empirical gap in understanding the acculturation–professional boundary (5–7).

We use the term *liminal bridge* to describe a transitional developmental zone in which recruits occupy an in-between identity state—no longer mere observers, yet not fully inducted teachers. Within occupational socialisation theory, such liminal experiences can accelerate, disrupt, or transform the movement from acculturation into professional socialisation by exposing recruits to authentic organisational norms and expectations. Singapore's Contract Teaching Stint (CTS) is uniquely positioned to illuminate this gap. CTS is a mandatory period of paid employment in schools undertaken before enrolment in the National Institute of Education. During this stint, recruits assume authentic teaching and co-curricular responsibilities while navigating mentorship, school leadership, and evaluation language. CTS can thus be theorised as a liminal overlap between acculturation and professional socialisation. If so, the quality of the CTS experience (CTSE) may be the hinge through which Initial Socialised Beliefs (ISB) are transformed into early Commitment to Teach (CTT).

Similar early-entry or pre-service employment schemes exist internationally, such as the School Direct and Teach First pathways in the United Kingdom, residency-based teacher education in the United States, and practicum intensives in Australia. These initiatives share the intent of bridging pre-entry beliefs with professional preparation through authentic school immersion, highlighting the broader relevance of Singapore's CTS model (8–11).

Guided by OST, this mixed-methods study asks four questions: (RQ1) how ISB, CTSE, and CTT are related; (RQ2) whether CTSE mediates the ISB–CTT relation, accounting for age and stint duration; (RQ3) whether these constructs vary by gender or programme (PGDE vs. DipPE); and (RQ4) how recruits explain the mechanisms through which CTS shapes commitment. Based on these research questions we generated four hypotheses: (H1) ISB would positively predict CTT; (H2) CTSE would partially mediate the ISB → CTT relationship; (H3) gender and programme differences would be small or non-significant; and (H4) qualitative explanations would align with and help interpret the ISB → CTSE → CTT pathway.

The study contributes (i) a conceptual clarification of OST's acculturation-professional bridge using a system-level, pre-entry stint, and (ii) practice-proximal guidance for mentoring, early pedagogical “wins”, and structured orientation in PE departments. Occupational socialisation theory (OST) provides a dominant lens for examining how teachers are shaped by multiple, interacting contexts across their careers. Originating in Lawson's work (3) and later elaborated by Templin and Schemp (12), OST differentiates among acculturation (prior experiences as pupils), professional socialisation (university and preservice preparation), and organisational socialisation (immersion in schools). These phases do not proceed sequentially; instead, they interact in complex ways. Research in physical education teacher education (PETE) has shown that the cultural traditions and micropolitics of schools often “wash out” the innovations introduced during university coursework (2, 5, 13). Conversely, supportive mentors and collegial school environments can sustain and amplify novices' willingness to try new pedagogies (14). Within this frame, identity work—the ongoing process of becoming and feeling like a teacher, rather than merely performing the role—has emerged as central to commitment, efficacy, and long-term retention (5, 9).

The Singapore CTS represents an unusual case for applying OST. Unlike typical preservice structures, CTS embeds candidates in schools before formal coursework, thereby accelerating exposure to organisational dynamics such as leadership expectations, evaluation logics, workload rhythms, and collegial norms. We theorise CTS as a hybrid, liminal phase that compresses OST's multiple strands: recruits are acculturated by their prior student experiences yet simultaneously immersed in authentic teaching roles before engaging professional coursework. This distinctive positioning makes CTS a plausible mechanism through which pre-entry beliefs are either reinforced or reshaped into commitments to teach. Emerging scholarship on early induction underscores that relational mentoring, opportunities for early classroom “wins”, and tangible skill acquisition (e.g., routines, safety management, planning exemplars) are crucial for identity consolidation. In contrast, workload shocks and bureaucratic demands often erode enthusiasm.

Against this backdrop, the present study advances three propositions: that CTS quality mediates the association between pre-entry beliefs and commitment, that mentoring climates explain observed links, and that evaluation and workload cues shape heterogeneity across recruits. Mediation was expected because OST posits that beliefs shape intentions primarily through contextualised, immersive experiences that reinforce or recalibrate those beliefs in early school settings. In doing so, the study positions CTS as a key bridging construct between acculturation and professional socialisation within PE teacher education (14, 15).

## 2 Methods

This study employed a convergent parallel mixed-methods design in which quantitative and qualitative data were collected during the same survey phase immediately following the CTS. Quantitative measures tested hypothesised relationships among ISB, CTSE, and CTT, while qualitative prompts elicited participants' explanations of how CTS shaped their beliefs and professional intentions. This design was selected because it allows complementary strands, statistical estimation of relationships, and interpretive exploration of mechanisms to address the research questions more comprehensively than either approach alone. Integration strengthened validity by using joint displays that aligned statistical pathways with explanatory qualitative mechanisms. This design goes beyond descriptive triangulation by demonstrating conceptual convergence across the two strands.

Both strands were implemented in a single data-collection wave; integration occurred during interpretation through joint displays that aligned statistical pathways (e.g., ISB → CTSE → CTT) with qualitative mechanisms (e.g., mentor scaffolds, workload balance). This structure ensures methodological transparency and supports replication.

### 2.1 Participants and recruitment

A census invitation was issued to the full intake (*N* = 103). Seventy-nine student teachers participated (response rate 76.7%; PGDE = 48; DipPE = 31; 61 male, 18 female). Inclusion criteria were: (i) completion of the CTS prior to enrolment, and (ii) current registration in either the PGDE or DipPE route. There were no exclusion criteria. Recruitment took place during the first programme week via a QR code link to the online information sheet and questionnaire. Participation was voluntary, uncompensated and anonymous. To understand generalisability, we compared respondents to cohort administrative tallies on gender and programme; descriptive deviations were minor and are discussed in the Results as context rather than as formal non-response tests.

### 2.2 Procedure

Participants provided consent before completing a 10–12 min anonymous survey on the institutional platform, which collected demographics, three multi-item constructs on ten-point response scales, and two open-ended questions regarding CTS experiences and preparation needs.

### 2.3 Measures

The survey instrument, designed for this study and informed by occupational socialisation theory (4, 5) and the CTS context, collected demographic information and measured three primary constructs using 10-point Likert-type scales. ISB operationalised pre-entry beliefs, CTSE operationalised induction experiences during CTS, and CTT captured commitment-related intentions. Sample items for each scale are provided below.

#### 2.3.1 Initial socialised beliefs (ISB)

ISB (10 items) measured recruits’ recalled beliefs about the purpose of PE, their anticipated role, and expected challenges before beginning CTS. *Sample item*: “I felt confident I could manage a PE class before starting CTS”.

#### 2.3.2 CTS experience (CTSE)

CTSE (5 items) assessed mentoring quality, opportunities for guided practice, workload realism, and departmental expectations. *Sample item*: “My mentor provided useful feedback that helped improve my teaching”.

#### 2.3.3 Commitment to teach (CTT)

CTT (5 items) measured participants’ intention to join and remain in the teaching profession. *Sample item*: “I intend to join the national teaching service after completing my programme”.

All items were adapted from OST-aligned instruments used in earlier PETE studies, expert-reviewed for clarity by PE faculty, and pilot-tested with a small group of preservice teachers to ensure comprehensibility. A complete list of items is provided in Supplementary Appendix A.

### 2.4 Data management and protection

Data were collected on a secure institutional survey platform, exported to encrypted, access-controlled institutional storage, and de-identified before analysis. No direct identifiers were collected; any indirect identifiers in open-ended responses were removed during cleaning. Data and materials will be retained for five years under institutional policy and Singapore's Personal Data Protection Act (PDPA).

### 2.5 Data preparation

We screened for completeness, out-of-range values, and patterned responding, and scored composites so higher values indicate stronger endorsement. Internal consistency is reported using Cronbach's α and McDonald's ω with 95% confidence intervals. Missing data were minimal (<4%) and were handled using listwise deletion for consistency across models. Outliers were screened using ±3 SD thresholds and Cook's distance; no cases exceeded decision cut-offs. (Item–level descriptive statistics and item–total ranges are provided in Supplementary Appendix B).

### 2.6 Quantitative analysis

Closed-ended responses were analysed using Python (v3.12.11) with the *pandas* library for data manipulation, and the *pingouin* and *factor_analyzer* libraries for statistical modelling. Diagnostic checks confirmed acceptable linearity, homoscedasticity, and normality of residuals. Given that all constructs were measured on ten-point response scales, the indicators approximate continuous distributions suitable for parametric analysis. Pearson correlations and linear mediation models are robust to minor deviations from normality, making them appropriate for the present analyses. The analytic steps include:

*Psychometric checks* assessed sampling adequacy with the Kaiser–Meyer–Olkin (KMO) index and Bartlett's test of sphericity. An Exploratory Factor Analysis (EFA) conducted using maximum likelihood with Promax (oblique) rotation, given correlated constructs. Items were scored on ten-point scales and treated as approximately continuous. Factor retention combined scree inspection with theory-congruent solutions, examined 2–4-factor solutions and selected the most parsimonious, interpretable model. Items were retained when loading ≥ .40 on the target factor with no salient cross-loadings (Δ ≥ .20). We present factor loadings/communalities in Supplementary Appendix C and report α/ω (95% CIs) for each composite in the main text/table.

*Descriptives and associations (RQ1)* report means/SDs and Pearson correlations with 95% CIs among ISB, CTSE and CTT, plus Age and CTS duration.

*Mediation test (RQ2)* estimated a regression-based mediation with bias-corrected bootstrapping (5,000 resamples) to test the indirect effect of ISB on CTT via CTSE, controlling for Age and CTS duration. We report a, b, c, c′ paths, ΔR^2^, unstandardised effects with 95% CIs, and the indirect effect. (Model syntax and diagnostics in Supplementary Appendix D).

*Group differences (RQ3)* analysed through Welch's *t*-tests (Satterthwaite df) used to compare mean scores by gender and programme (PGDE vs. DipPE), reporting Hedges' g with 95% CIs. Given the smaller female subsample, we interpret effects cautiously.

Assumptions and diagnostics (outliers, linearity, homoscedasticity, multicollinearity) were inspected; where assumptions were strained, with robust statistics (e.g., Welch's t) and bootstrap CIs to avoid over-interpretation of *p*-values.

### 2.7 Qualitative analysis and integration

Reflexive thematic analysis was employed in the qualitative study. One analyst coded all responses; themes were iteratively developed, reviewed against the dataset, and named according to established boundary rules. The final codebook for Q21 & Q22, which includes theme definitions, inclusion criteria, and salience indicators, is presented in Supplementary Appendices E, F. An audit trail (versioned codebook, logs, and memos) is maintained; inter-coder coefficients were not calculated, consistent with a reflexive stance. Credibility was supported via peer debrief and negative-case analysis, integrated strands, a joint display aligning statistical signals (e.g., ISB→CTSE→CTT pathway) constructed with explanatory themes (mentor scaffolds, pedagogical craft, work realities) and implications for mentoring and programme design.

### 2.8 Ethics

The study was conducted in accordance with the Nanyang Technological University Institutional Review Board (NTU IRB). Approval was obtained prior to recruitment (Ref: IRB-2025-465). Proceeding to the questionnaire indicated informed consent. Data were anonymised and stored on encrypted, access-controlled institutional drives.

## 3 Results

This section is organised into three parts: (a) quantitative findings, including preliminary sample analyses, descriptive statistics, the primary mediation and group comparison models addressing the research questions; (b) qualitative findings from the open-ended survey responses; and (c) an integrated discussion synthesising insights from both strands of data.

### 3.1 Preliminary analyses

The final sample consisted of 79 student teachers, representing a 76.7% response rate. A comparison of respondent demographics against the total cohort data revealed that respondent proportions were comparable to cohort distributions (gender, programme). In Table 1, the PGDE cohort was, on average, older than the Diploma (PE) cohort. The average duration of CTS was comparable between the two programmes. Effect sizes for age and CTS duration differences between programmes were small (Hedges' g < .25), indicating good demographic comparability across groups.

**Table 1:** Participant demographics.

Characteristic	*n*	%	*M*	*SD*	Range
Gender					
Male	61	77.2	–	–	–
Female	18	22.8	–	–	–
Programme					
PGDE	48	60.8	–	–	–
DipPE	31	39.2	–	–	–
Age (years)					
PGDE	–	–	30.6	6.0	25–53
DipPE	–	–	27.1	4.4	21–44
CTS Duration (months)	–	–			
PGDE	–	–	9.0	3.4	5–18
DipPE	–	–	8.6	3.4	4–17

PGDE, postgraduate diploma in education; DipPE, diploma in physical education. Total Sample (*N* = 79).

### 3.2 Measurement properties

Sampling was suitable for factor analysis: the KMO of 0.733 indicates adequate shared variance among items for common-factor modelling, and Bartlett's test (*χ*^2^ = 725.28, *p* < .001) rejects the null of an identity correlation matrix, supporting factorability. We used parallel analysis, which outperforms eigenvalues greater than one and scree plots alone to select dimensionality; it indicated a two-factor solution at the item level. Given our 10-point response format (approximately continuous indicators) and the expectation that the latent constructs are correlated (ISB, CTSE, and CTT are theoretically linked), we estimated an exploratory factor analysis (EFA) using maximum likelihood and Promax (oblique) rotation. For construct scores used in subsequent analyses, we followed the study's *a priori* conceptual mapping: ISB = Q1–Q10, CTSE = Q11–Q15, CTT = Q16–Q20. This preserves theoretical content coverage while allowing the EFA to serve as a structure check rather than a *post-hoc* item re-allocation. Internal consistency was acceptable to good for all composites (Table 2), supporting their use in correlation and mediation models. The EFA served as a structure check rather than a scale-development procedure, confirming the coherence of the *a priori* theoretical mapping (ISB: Q1–Q10; CTSE: Q11–Q15; CTT: Q16–Q20). Primary loadings were strong (ISB items .56–.82; CTSE/CTT items .61–.88), and cross-loadings were minimal (Δ ≥ .25), supporting discriminant but related constructs consistent with occupational socialisation theory.

**Table 2:** Descriptive statistics, reliabilities, and correlations for key variables.

Variable	M (SD)	1	2	3	4	5
1. Initial Socialised Beliefs (ISB)	7.67 (1.21)	(.77)				
2. CTS Experience (CTSE)	8.80 (1.18)	.43*	(.75)			
3. Commitment to Teach (CTT)	8.68 (1.20)	.42*	.59*	(.88)		
4. Age (years)	29.2 (5.69)	−.05	.10	−.11	–	
5. CTS Duration (months)	8.8 (3.36)	.09	−.04	−.09	.00	–

*N* = 79.

* *p* < .01. Reliability coefficients (Cronbach's α) are diagonal in parentheses. Scores are on a ten-point scale.

### 3.3 Descriptive statistics and bivariate correlations

All correlations were computed using listwise deletion and are based on *N* = 79. ISB related moderately to both CTSE (r = .42) and CTT (r = .42); CTSE correlated with CTT (r = .59). Associations with Age and CTS duration were small and non-significant. Descriptive statistics and bivariate correlations for the three primary constructs are presented in Table 2. In relation to RQ1, neither age nor the duration of the CTS was significantly associated with student teachers' initial socialised beliefs.

### 3.4 Addressing the main research questions

To test the hypothesis that the CTS experience mediates the relationship between initial beliefs and commitment, we performed a regression-based mediation analysis, controlling for participant **Age** and **CTS Duration**. Bias-corrected bootstrapping (5,000 resamples) indicated a significant indirect effect of ISB on CTT via CTSE [indirect = 0.22, 95 % CI (0.11, 0.38)]. The direct effect of ISB on CTT remained small but significant after accounting for CTSE (c′ = 0.20, *p* = .048), consistent with partial mediation (a = 0.42; b = 0.62; total c = 0.42). Mediator and outcome models explained R² values of .193 and .414, respectively. The results, generated from a bias-corrected bootstrapping procedure with 5,000 resamples, are presented in Table 3.

**Table 3:** Mediation analysis of the effect of initial beliefs on commitment to teach via CTS experience.

Path	Effect	Coefficient	SE	*p*-value	95% CI
ISB → CTSE	Path a	0.42	0.10	<.001	[0.22, 0.62]
CTSE → CTT	Path b	0.62	0.09	<.001	[0.43, 0.80]
Total Effect (ISB → CTT)	Path c	0.42	0.10	<.001	[0.21, 0.62]
Direct Effect (ISB → CTT)	Path c'	0.20	0.10	.048	[0.00, 0.39]
Indirect Effect (a*b)	Mediation	0.22	0.07	<.001	[0.11, 0.38]

*N* = 79. ISB, initial socialised beliefs; CTSE, CTS experience; CTT, commitment to teach; CI, confidence interval, generated from 5,000 bootstrap samples.

Standardised coefficients supported the same pattern: a = .41, b = .58, c = .39, and c′ = .18, with a moderate indirect effect (ab = .24). These standardised effects reinforce the partial mediation interpretation. The indirect effect was moderate in magnitude, whereas the residual direct effect was small, further supporting partial rather than full mediation.

Only CTSE differed by gender—Male > Female—with a large effect (9.01 ± 1.07 vs. 8.09 ± 1.26), Welch t(24.70) = 2.80, *p* = .010, Hedges' g = 0.81 [0.28, 1.35]; all other gender and programme contrasts were non-significant. No other gender or programme differences were significant, as detailed in Table 4. Results were robust to multiple-comparison correction using the Holm–Bonferroni method; only the gender difference in CTSE remained statistically significant.

**Table 4:** Group differences by gender and programme.

Construct	Group comparison	Group 1 *M (SD)*	Group 2 *M (SD)*	Welch t(df)	*p*-value	Hedges’ g [95% CI]
Initial Socialised Beliefs (ISB)	Male (*n* = 61) vs. Female (*n* = 18)	7.69 (1.28)	7.58 (0.97)	t(36.24) = 0.39	0.698	0.089 [−0.431, 0.610]
CTS Experience (CTS-E)	Male (*n* = 61) vs. Female (*n* = 18)	9.01 (1.07)	8.09 (1.26)	t(24.70) = 2.80	0.010*	0.812 [0.276, 1.349]
Commitment to Teach (CTT)	Male (*n* = 61) vs. Female (*n* = 18)	8.72 (1.28)	8.52 (0.89)	t(40.07) = 0.76	0.452	0.166 [−0.355, 0.687]
Initial Socialised Beliefs (ISB)	PGDE (*n* = 48) vs. DipPE (*n* = 31)	7.79 (1.09)	7.47 (1.38)	t(53.47) = 1.09	0.281	0.261 [−0.188, 0.710]
CTS Experience (CTS-E)	PGDE (*n* = 48) vs. DipPE (*n* = 31)	8.82 (1.24)	8.77 (1.08)	t(70.32) = 0.18	0.854	0.041 [−0.406, 0.488]
Commitment to Teach (CTT)	PGDE (*n* = 48) vs. DipPE (*n* = 31)	8.59 (1.16)	8.81 (1.27)	t(60.05) = −0.78	0.438	−0.182 [−0.630, 0.267]

Welch's *t*-tests reported with Satterthwaite degrees of freedom. Effect sizes are Hedges’ g with 95% confidence intervals (pooled SD with small-sample correction).

* *p* < .05. Scores are on a ten-point scale.

### 3.5 Qualitative findings

Across the dataset, approximately 70%–80% of participants contributed statements coded into each of the four major themes. We used a reflexive thematic analysis (interpretivist stance) (16) to explain *how* the CTS shaped beliefs and intentions. Memos, peer debriefs, and negative-case probing were used to refine candidate mechanisms and boundary conditions. Four explanatory themes follow, with cross-case contrasts and a compact typology.

#### 3.5.1 Theme 1: CTS foregrounds classroom management and routines

***Claim***. Participants entered with performance-centred images of PE but left prioritising classroom management and routine-building.

##### 3.5.1.1 Mechanism

Daily lesson realities made *routines and behaviour* the immediate currency of effectiveness.

##### 3.5.1.2 Evidence

“There was a lot more classroom management and non-PE lessons related duties than expected”. (TS40, PGDE)

“Better understanding of classroom management”. (TS02, PGDE)

“It was working with students to change their behaviour and seeing that change in them”. (TS72, DipPE)

“…you truly see how students behave during unstructured (in a sense) time”. (TS73, DipPE)

##### 3.5.1.3 Interpretation

These accounts show subjective warrants being re-weighted toward routines, transitions, and behaviour shaping. CTS functions as an early reality test that narrows the “activity first” lens many carry from pre-entry acculturation. This emphasis on classroom routines aligns with the positive ISB → CTSE association (path a = .42), suggesting that recruits with stronger initial beliefs reported greater gains in their CTS when routine-building was prioritised.

##### 3.5.1.4 Policy Implication

Programme language and assessment rubrics should make routinisation and behaviour shaping *explicit* success criteria during CTS.

#### 3.5.2 Theme 2: mentor micro-cycles are the proximal mechanism of efficacy gain

##### 3.5.2.1 Claim

Where mentors engineered *observe → precise feedback → re-teach* loops, efficacy rose quickly.

##### 3.5.2.2 Mechanism

Micro-cycles combined *mastery experiences* with *credible feedback* in the same lesson arc.

##### 3.5.2.3 Evidence

“Conducting lessons and having mentors monitoring and giving feedbacks”. (TS25, PGDE)

“My mentoring sessions, because my mentor really challenged my views on lessons preparations and my own readiness and confidence”. (TS54, DipPE)

“My mentor was compassionate and supportive, always encouraging me…” (TS47, PGDE)

“Maybe 1–2 weeks of observing my mentor … regular check-ins with my mentor about how to improve on my teaching”. (TS23, PGDE)

##### 3.5.2.4 Interpretation

Participants tie confidence not to generic encouragement but to *structured* cycles that create immediate, repeatable wins (e.g., transitions, voice, positioning). The desire for more observation and check-ins signals where the mechanism is missing. The structured observe–feedback–reteach cycles described in this theme map closely onto the strong CTSE → CTT pathway (path b = .62), demonstrating how guided mentoring sequences directly support the development of professional commitment.

##### 3.5.2.5 Policy Implication

Specify a minimum mentoring pattern during CTS: at least one micro-cycle per observed segment (observe → cue → re-teach).

#### 3.5.3 Theme 3: work design and paperwork trigger identity work

##### 3.5.3.1 Claim

Administrative demands and time pressure shift identity towards sustainable teaching routines, sometimes with strain.

##### 3.5.3.2 Mechanism

Role overload and limited access to timely help force pragmatic reprioritisation.

##### 3.5.3.3 Evidence

“The amount of “paperwork” that had to be done”. (TS55, DipPE)

“Having someone you could contact to talk to about fear and new situations … sometimes it can be difficult as your mentor may be busy, and other teachers are seemingly swamped with work as well”. (TS49, DipPE)

##### 3.5.3.4 Interpretation

Where paperwork and limited access to mentors coincide, identity shifts are reactive rather than developmental. The same mechanism that builds efficacy (Theme 2) stalls without time and access. Participants clearly differentiated between administrative workload (e.g., paperwork, CCA duties, school events) and pedagogical workload (e.g., lesson planning, assessment, safety routines), suggesting that future research should measure these domains separately given their distinct effects on identity formation.

##### 3.5.3.5 Policy Implication

Protect brief mentor access windows and provide routine “admin packs” (templates, trackers) to reduce cognitive load while routines consolidate.

#### 3.5.4 Theme 4. Boundary conditions and variation in trajectories

##### 3.5.4.1 Claim

Not all entrants needed more support; some reported sufficiency, others flagged structured preparation as helpful.

##### 3.5.4.2 Mechanism

Prior experience or strong school supports can substitute for missing micro-cycles; conversely, structured pre-CTS inputs can prime them.

##### 3.5.4.3 Evidence

“The support is very good”. (TS27, PGDE)

“I received adequate support”. (TS36, PGDE)

“None needed. I was prepared”. (TS37, PGDE)

“The Introduction to Teaching Programme  … would better prepare us … before commencing our CTS”. (TS22, PGDE)

##### 3.5.4.4 Interpretation

The efficacy mechanism is *conditional*: prior readiness and local school design can offset gaps; structured pre-CTS orientation strengthens the ramp for those without school-based experience.

##### 3.5.4.5 Policy Implication

Pair CTS with a concise pre-entry “routines & micro-cycles” primer; keep it targeted rather than broad.

### 3.6 Cross-case contrasts (programme route & mentoring)

Both programme routes emphasised management and routines; PGDE participants more often asked for **classroom management knowledge** (e.g., TS02), while DipPE participants explicitly described **behaviour change** in pupils (e.g., TS72–TS73). Across routes, the decisive differentiator was **mentor practice**: where observation, feedback, and re-teaching were present, confidence rose (TS25, TS54, TS47); where mentor access was constrained, gains lagged (TS49).

In addition, we observed three recurrent trajectories:

1. **Affirmers**: CTS *confirmed* a teaching-orientation; minor adjustments only. *Trigger:* Prior school-based experience; supportive routines.
2. **Recalibrators**: Shifted from performance- to teaching-orientation. *Trigger:* Mentor micro-cycles + early role-set contact.
3. **Dissonance-resolvers**: Persistent coaching identity or ambivalence. *Trigger:* Absent/weak mentoring; club commitments; custodial school culture.

Based on coded frequencies, approximate proportions were: Affirmers (20%–25%), Recalibrators (55%–60%), and Dissonance-resolvers (15%–20%). Table 5 below illustrates a compact typology of CTS trajectories.

**Table 5:** A compact typology of CTS trajectories.

Trajectory	Trigger(s)	Proximal mechanism	Outcome in beliefs/identity	Illustrative excerpt
Affirmers (∼some)	Prior school exposure; clear routines	Confirmation via routine success	Teaching orientation affirmed; low strain	“None needed. I was prepared”. (TS37, PGDE)
Recalibrators (many)	Daily lesson realities; mentor micro-cycles	Mastery + credible feedback	Shift to teaching orientation; efficacy gains	“Conducting lessons… mentors… giving feedbacks”. (TS25, PGDE)
Dissonance-resolvers (few)	Admin load; limited mentor access	Overload; ad-hoc help	Pragmatic shift with strain/mixed identities	“…mentor may be busy… others are swamped…” (TS49, DipPE)

### 3.7 Integrative takeaways

Across cases, CTS works not merely as *exposure* but as an engine of recalibration: everyday routines and behaviours become central; mentor microcycles convert feedback into efficacy; and work design conditions whether identity shifts are durable or draining. Designing CTS around these mechanisms and their boundary conditions is the most straightforward path to stronger, earlier impacts on entrants' professional identities.

Across themes, the CTS appeared to convert initial inclination into commitment through three proximal mechanisms: mentor scaffolds (observation, calibrated feedback, gradual release), early student-centred wins, and concrete craft learning (planning, management, safety). These mechanisms plausibly raise CTSE, cohering with the CTSE–CTT association (r = .59) and the significant ISB→CTSE→CTT indirect effect (0.22); the small residual direct path (c′ = 0.20) suggests some commitment precedes the stint. At the same time, work-reality pressures (administration, time, CCA/events) emerged as dampeners of CTSE, clarifying why the pattern is partial rather than full mediation. The joint display consolidates these links and anchors them in quotes that show both convergence and exceptions (Supplementary Appendix G; see also Tables 2, 3).

The convergence is clear: the quantitative pattern—ISB relates to CTT chiefly via CTSE (indirect = 0.22; CTSE–CTT r = .59; residual c′ = 0.20)—is explained by narratives of mentor scaffolds, early student “wins”, and concrete craft learning during the stint. These mechanisms give the statistical paths practice-proximal meaning and indicate where departments can act (Tables 2, 3; Supplementary Appendix C).

Complementarity appears in pre-CTS levers (shadowing, planning exemplars, PE-specific routines) that plausibly lift CTSE upstream; divergence is most visible where work realities depress CTSE despite confident beliefs, aligning with partial—rather than full—mediation. The joint display (Supplementary Appendix C) marks these linkages explicitly and retains counter-examples so the qualitative strand illuminates rather than retrofits the statistics.

## 4 Discussion

The mediation analysis demonstrated that Initial Socialised Beliefs influenced Commitment to Teach primarily through the CTS Experience, with a moderate indirect effect (ab = .22) and only a small residual direct effect. This finding establishes the quantitative foundation for the integrated interpretation that follows, in which qualitative mechanisms such as mentoring structures, early instructional success, and workload rhythms explain how CTS quality shapes recruits' emerging identities and commitment.

Although CTS occurred prior to the measurement of all variables, reciprocal influence cannot be fully ruled out. It is possible that individuals with stronger pre-existing commitment retrospectively evaluated their CTS more positively. Longitudinal research is required to determine whether the observed associations reflect unidirectional or bidirectional processes.

The partial mediation pattern (ISB → CTSE → CTT), with only a modest direct pathway, highlights the pivotal role of stint quality in converting prior beliefs into longer-term professional commitment. While initial subjective warrants about teaching PE retain some influence, they are enacted mainly and reshaped through authentic school experiences. The moderate CTSE–CTT link (r = .59) highlights how scaffolded mentoring, gradual release to co-teaching, targeted feedback, and early instructional success can transform predispositions into a durable identity. Situated within occupational socialisation theory, these findings extend the notion of “subjective warrant” (3, 4) by illustrating warrant-testing in authentic contexts, with confirmation, recalibration, and resistance reflecting dynamic, dialectical encounters between individuals and organisations.

Importantly, participants also drew attention to stressors—administrative routines, CCAs, and duty cycles—that, without structured support, can constrain identity development and motivation (17) and broader induction research (18, 19). Conceptually, the CTS represents a pre-induction phase that bridges early acculturation and formal professional socialisation. Practically, it points to three actionable school-level levers: (i) deliberate mentor pairing and protected observation/feedback time; (ii) engineered sequences enabling early teaching success; and (iii) straightforward onboarding to workload and CCA systems. These insights affirm that stint design is not a passive placement process but a critical arena where belief is tested, supported, and transformed into professional commitment. While modest gender differences in stint perceptions suggest future inquiry, the study demonstrates that structured CTS can shape durable professional identity in early-career PE teachers.

### 4.1 Theoretical contribution

Our analysis extends occupational socialisation theory in four ways:

1. **Recalibration timing:** It identifies the acculturation–professional seam as the point where subjective warrants are most susceptible to change during early, authentic experience.
2. **Proximal mechanism:** It positions mentor microcycles as the primary pathway through which efficacy and identity strengthen, aligning with mastery-based theories of teacher development.
3. **Work design effects:** It shows how administrative and pedagogical workload shape whether identity shifts are stabilising or destabilising during early school immersion.
4. **Mechanism-oriented OST:** It advances OST from describing phases to specifying mechanisms, thresholds, and organisational conditions that shape early professional commitment.

Although the present study is situated in Singapore, its findings have international applicability. The mechanisms identified, structured mentoring, workload balance, and early mastery experiences, resonate with teacher-induction and residency models in the United Kingdom, the United States, and Australia, which similarly emphasise guided practice and scaffolded responsibility (8–11). Designing early-career placements around these levers can therefore inform global policy conversations on sustaining teacher commitment.

Future research could examine longitudinal trajectories across the full teacher-preparation pipeline or assess how the intensity and structure of mentoring interactions predict long-term retention. Comparative studies across systems with differing induction policies would also clarify which contextual factors amplify or dampen the belief-to-commitment pathway.

### 4.2 Limitations

Given that participants reported their pre-CTS beliefs retrospectively, responses may be influenced by recall error and social desirability, warranting cautious interpretation. While the indirect-effect model is consistent with mediation, temporal sequencing of ISB, CTSE, and CTT cannot be confirmed. Findings are based on a single cohort of PE student teachers in Singapore and may not generalise more broadly. The qualitative strand was coded reflexively by one analyst to maintain interpretive coherence, which may introduce researcher bias. Despite this limitation, a single-coder approach aligns with a reflexive thematic analysis stance, which emphasises analytic depth, interpretive coherence, and the researcher's active role in meaning-making rather than inter-coder agreement metrics. Credibility was supported through multiple safeguards: analytic memos documenting decision trails, regular peer debriefing with colleagues to interrogate theme boundaries, and deliberate searching for negative or disconfirming cases. These practices enhanced transparency and reflexivity while ensuring that themes remained grounded in the data rather than in coder consensus.

## 5 Conclusion

Across this cohort, CTS quality emerged as the central mechanism linking initial beliefs to later commitment. Mentor pairing, structured feedback loops, early instructional success, and clear workload scaffolding were the most actionable levers that converted predispositions into durable professional identity. These findings demonstrate that CTS is not simply an exposure period but a pivotal developmental phase that shapes teacher commitment.

Practically, the findings align with broader induction studies, which demonstrate that structured mentoring and organisational supports promote retention, while weak induction heightens vulnerability to workload “reality shock” (20–26). Participants' calls for strategic mentor pairing, phased release for teaching, and straightforward onboarding to workload and CCA duties highlight tractable levers for schools. Conceptually, CTS functions as a bridge between acculturation and professional socialisation. When designed deliberately, it provides a system-specific mechanism to consolidate commitment and transform pre-entry intention into a durable professional identity.

This study moves beyond descriptive accounts of early school experience to identify *how* beliefs change. Role-set contact during CTS recalibrates subjective warrants; guided mentoring sequences are the proximal pathway to classroom-management efficacy; and work design governs whether identity shifts take root or fray. These mechanisms specify timing, agents, and thresholds within occupational socialisation. Designing CTS around these levers—briefing mentors to run micro-cycles, protecting short debrief windows, and providing routine/admin scaffolds—offers a practical route to stronger, earlier impacts on entrants' professional identities and, ultimately, classroom stability.

Future research should employ longitudinal tracking or experimental manipulation of mentoring intensity to more definitively establish the causal sequencing among ISB, CTSE, and CTT.

## Data Availability

The raw data supporting the conclusions of this article will be made available by the authors, without undue reservation.
