# Supplementary material for: Contract teaching as a liminal bridge: how pre-entry beliefs become commitment in PE teacher socialisation
Source: Front Sports Act Living. 2025 Dec 18;7:1719826. doi: 10.3389/fspor.2025.1719826 (PMC12756359; doi:10.3389/fspor.2025.1719826)
Supplement: Supplementary file 1 [file Datasheet1.docx]

# Appendix A.

## Full item list (ISB, CTSE, CTT)

### 26-Item Survey Instrument

For Items Q1 to Q20, Participants will respond using a 10-point Likert scale (1 = strongly disagree, 10 = strongly agree).

Demographic Data:

1. Gender (1= Male, 2= Female)
2. Age (open-ended)
3. Programme Type (1= PGDE, 2= DipEd, 3=BEd/BSc)
4. Duration of Contract Teaching Stint (CTS) in months (open-ended)

Intrinsic Motivation:

1. I have always enjoyed participating in physical activities and sports.
2. Teaching PE aligns with my personal interests and passions.
3. I chose PE teaching because I want to influence children’s lives positively.

Extrinsic Motivation:

1. Job security was an important factor in my decision to become a PE teacher.
2. Family or societal expectations influenced my choice to become a PE teacher.
3. Potential career advancement opportunities influenced my decision to teach PE.

Initial Beliefs about Teaching:

1. My experiences as a student in PE classes positively influenced my decision to teach PE.
2. Inspirational PE teachers/coaches significantly shaped my teaching beliefs.
3. I believed teaching PE would be straightforward and enjoyable based on my past experiences.
4. Coaching sports teams initially seemed more appealing to me than classroom PE teaching.

CTS Experience:

1. My CTS experience provided a realistic preview of the workload and responsibilities of a PE teacher.
2. I received consistent and constructive feedback from my mentor teacher(s) during the CTS.
3. I felt supported by my peers and colleagues during my CTS.
4. My CTS experience aligned with what I expected teaching PE in schools would be like.
5. My CTS challenged some of my initial assumptions about the realities of teaching PE.

Commitment to Teach:

1. My CTS experience increased my commitment to becoming a PE teacher.
2. I feel confident about teaching PE after the CTS experience.
3. My teaching commitment was strengthened by interactions with students during the CTS.
4. After the CTS, I am more likely to remain a PE teacher long-term.
5. I would recommend teaching PE to others based on my CTS experience.

Open-Ended Survey Questions

Q21. In your own words, describe the most impactful experience you had during your Contract Teaching Stint (CTS). How did it influence your perception of teaching PE?

Q22. Reflecting on your CTS, what support or preparation would you have liked to have received before starting the stint?
