# Supplementary material for: Contract teaching as a liminal bridge: how pre-entry beliefs become commitment in PE teacher socialisation
Source: Front Sports Act Living. 2025 Dec 18;7:1719826. doi: 10.3389/fspor.2025.1719826 (PMC12756359; doi:10.3389/fspor.2025.1719826)
Supplement: Supplementary file 2 [file Datasheet2.docx]

# Appendix B.

## Item-level descriptives and item–total correlations

### Method.

For each item (Q1–Q20), we report mean (M), standard deviation (SD), skewness, kurtosis, and corrected item–total correlation (CITC) within its scale, alongside Cronbach’s alpha if item deleted. Scales: ISB = Q1–Q10; CTSE = Q11–Q15; CTT = Q16–Q20. CITC uses listwise deletion within each scale.

**Table B1.**

ISB (Q1–Q10)

| **Item** | **M** | **SD** | **Skew** | **Kurtosis** | **CITC** | **α if deleted** |
| --- | --- | --- | --- | --- | --- | --- |
| Q1 | 9.405 | 0.954 | −2.173 | 6.172 | 0.380 | 0.763 |
| Q2 | 9.089 | 1.200 | −2.091 | 7.374 | 0.559 | 0.747 |
| Q3 | 9.329 | 0.996 | −1.426 | 1.207 | 0.405 | 0.761 |
| Q4 | 8.152 | 2.082 | −1.257 | 1.743 | 0.358 | 0.760 |
| Q5 | 5.228 | 2.722 | 0.270 | −0.625 | 0.659 | 0.713 |
| Q6 | 6.392 | 2.677 | −0.380 | −0.537 | 0.506 | 0.740 |
| Q7 | 7.835 | 2.356 | −1.059 | 0.364 | 0.514 | 0.739 |
| Q8 | 7.848 | 2.143 | −0.696 | −0.239 | 0.441 | 0.749 |
| Q9 | 7.734 | 1.859 | −0.645 | 0.119 | 0.487 | 0.745 |
| Q10 | 5.671 | 3.066 | −0.124 | −1.130 | 0.328 | 0.778 |

**Table B2.**

CTSE (Q11–Q15)

| **Item** | **M** | **SD** | **Skew** | **Kurtosis** | **CITC** | **α if deleted** |
| --- | --- | --- | --- | --- | --- | --- |
| Q11 | 8.785 | 1.473 | −1.443 | 2.356 | 0.493 | 0.709 |
| Q12 | 9.013 | 1.691 | −2.192 | 4.748 | 0.636 | 0.653 |
| Q13 | 9.278 | 1.154 | −1.545 | 1.271 | 0.622 | 0.685 |
| Q14 | 8.658 | 1.804 | −1.555 | 2.014 | 0.485 | 0.712 |
| Q15 | 8.253 | 2.085 | −1.218 | 1.092 | 0.426 | 0.750 |

**Table B3.**

CTT (Q16–Q20)

| **Item** | **M** | **SD** | **Skew** | **Kurtosis** | **CITC** | **α if deleted** |
| --- | --- | --- | --- | --- | --- | --- |
| Q16 | 8.747 | 1.531 | −1.340 | 1.832 | 0.772 | 0.845 |
| Q17 | 8.291 | 1.478 | −0.618 | 0.095 | 0.664 | 0.870 |
| Q18 | 8.911 | 1.190 | −1.091 | 2.000 | 0.778 | 0.850 |
| Q19 | 8.899 | 1.446 | −1.514 | 2.046 | 0.727 | 0.855 |
| Q20 | 8.544 | 1.616 | −0.961 | −0.019 | 0.686 | 0.868 |
