# Supplementary material for: Contract teaching as a liminal bridge: how pre-entry beliefs become commitment in PE teacher socialisation
Source: Front Sports Act Living. 2025 Dec 18;7:1719826. doi: 10.3389/fspor.2025.1719826 (PMC12756359; doi:10.3389/fspor.2025.1719826)
Supplement: Supplementary file 3 [file Datasheet3.docx]

# Appendix C.

## EFA loadings and communalities

Method. Maximum likelihood EFA with promax rotation on 20 items (N = 79 complete cases). Parallel analysis retained 2 factor(s). KMO = 0.733; Bartlett’s χ² = 725.28, p = 1.56e-63.

Display rule. Primary loadings (largest absolute per row) ≥ .40 are bolded. A dagger (†) marks cross‑loadings where the non‑primary absolute loading ≥ .30.

| **Item** | **F1** | **F2** | **h²** |
| --- | --- | --- | --- |
| Q1 | 0.182 | 0.344 | 0.152 |
| Q2 | 0.254 | **0.491** | 0.305 |
| Q3 | 0.255 | 0.322 | 0.169 |
| Q4 | 0.016 | **0.417** | 0.174 |
| Q5 | 0.042 | **0.715** | 0.512 |
| Q6 | -0.100 | **0.633** | 0.410 |
| Q7 | -0.000 | **0.560** | 0.313 |
| Q8 | -0.033 | **0.542** | 0.295 |
| Q9 | -0.304† | **0.750** | 0.655 |
| Q10 | 0.027 | 0.365 | 0.134 |
| Q11 | 0.376 | 0.163 | 0.168 |
| Q12 | 0.339 | 0.268 | 0.187 |
| Q13 | **0.452** | 0.049 | 0.207 |
| Q14 | **0.718** | -0.090 | 0.523 |
| Q15 | 0.357 | 0.085 | 0.135 |
| Q16 | **0.901** | -0.155 | 0.835 |
| Q17 | **0.791** | -0.062 | 0.630 |
| Q18 | **0.949** | -0.141 | 0.921 |
| Q19 | **0.735** | -0.025 | 0.541 |
| Q20 | **0.730** | -0.096 | 0.542 |

Notes. Loadings are pattern coefficients. Signs reflect factor orientation and do not affect interpretation. Thresholds guide reporting; items are retained on theoretical grounds as specified in the a priori construct mapping.
