# Supplementary material for: Contract teaching as a liminal bridge: how pre-entry beliefs become commitment in PE teacher socialisation
Source: Front Sports Act Living. 2025 Dec 18;7:1719826. doi: 10.3389/fspor.2025.1719826 (PMC12756359; doi:10.3389/fspor.2025.1719826)
Supplement: Supplementary file 4 [file Datasheet4.docx]

**Appendix E.**

## Codebook — Q21: Most Impactful Aspect of the CTS (N = 79)

Frequencies are reported as descriptive indicators of salience to aid interpretation; we do not infer prevalence beyond this sample

**Table E1***.* Codebook — Q21.

| **Theme** | **Brief definition** | **Inclusion rules** | **Exclusion rules** | **Salience**  **n (%)** | **Exemplar extract (ID)** |
| --- | --- | --- | --- | --- | --- |
| Relational & organisational supports | Practical or affective support from mentors, heads of department, or the wider department that accelerates early success. | Observation windows; targeted feedback; co-teaching; pastoral care. | Generic “I felt supported” with no identified source or action; student praise (see Student engagement). | 18 (22.8) | “Receiving feedback on my strengths and areas for growth.” [TS13] |
| Teacher identity & confidence | Growing sense of fit or purpose; feeling like “a real teacher.” | Breakthroughs with a class; being trusted; recognition. | Student outcomes only (see Student engagement); administrative load. | 13 (16.5) | “Breakthrough with a challenging student… rewarding when he finally participated.” [TS34] |
| Student engagement & learning | Student enjoyment or progress as confirmation of purpose. | Positive responses; increased participation; learning milestones. | Planning or technique detail (see Pedagogical craft). | 12 (15.2) | “The students… telling me they love my lesson.” [TS56] |
| Pedagogical craft (planning, management, safety) | Concrete learning about lesson design, management, and safety. | Task progressions; routines; risk management; reflection on instructional moves. | General “support” without a craft element (see Relational). | 10 (12.7) | “A lot more classroom management… than expected.” [TS40] |
| Facilities & resources | Physical or logistical affordances and constraints shaping feasibility. | Space or equipment access; wet-weather plans; booking systems. | Time or co-curricular activity load (see Work realities). | 7 (8.9) | “Limited space changed how I set up activities.” [TS62] |
| Work realities (administration, time, CCA/events) | Non-instructional demands producing reality shock. | Form class; paperwork; event or CCA burden; timetable constraints. | Pedagogical routines (see Pedagogical craft). | 4 (5.1) | “Having to take a form class [was unexpected].” [TS76] |
| Inclusive practice (SEN) | Exposure to and strategies for diverse learners. | Adapting tasks; collaboration with SEN staff; use of aides. | Generic engagement without a SEN element. | 2 (2.5) | “Supporting students with special needs.” [TS28]; “Making PE inclusive… including SEN.” [TS21] |

Note. Multiple codes were permitted; percentages are calculated with N = 79. Quotes are anonymised, with minimal edits for clarity.
