# Supplementary material for: Contract teaching as a liminal bridge: how pre-entry beliefs become commitment in PE teacher socialisation
Source: Front Sports Act Living. 2025 Dec 18;7:1719826. doi: 10.3389/fspor.2025.1719826 (PMC12756359; doi:10.3389/fspor.2025.1719826)

# Appendix D.

## Mediation model syntax and diagnostics

*Model specification.* Single-mediator model with ISB as predictor (X), CTSE as mediator (M), and CTT as outcome (Y). Covariates: Age and CTS months in both mediator and outcome models. Effects estimated via non-parametric bootstrapping (5,000 resamples; percentile confidence intervals). Coefficients are unstandardised; R-squared is from standardised OLS models.

# *Equations.*

Mediator (CTSE): CTSE = a·ISB + c1·Age + c2·CTS_months + e_M
Outcome (CTT): CTT = b·CTSE + c′·ISB + d1·Age + d2·CTS_months + e_Y
Total effect: c = c′ + a·b; Indirect = a·b; Direct = c′

**Table D1.**

Mediation effects (bootstrap, 5,000)

| **Path / Effect** | **Coefficient** | **SE** | **p-value** | **95% CI (lower)** | **95% CI (upper)** | **Sig.** |
| --- | --- | --- | --- | --- | --- | --- |
| CTSE ~ ISB (a) | 0.418 | 0.101 | <.001 | 0.216 | 0.62 | Yes |
| CTT ~ CTSE (b) | 0.619 | 0.093 | <.001 | 0.433 | 0.804 | Yes |
| Total effect (c) | 0.42 | 0.103 | <.001 | 0.214 | 0.625 | Yes |
| Direct effect (c′) | 0.198 | 0.098 | 0.0481 | 0.002 | 0.394 | Yes |
| Indirect effect (a·b) | 0.222 | 0.07 | < .001 | 0.105 | 0.383 | Yes |

### *Model fit and variance explained.*

Mediator model R-squared = 0.193; Outcome model R-squared = 0.414

### *Diagnostics.*

Multicollinearity (VIF): mediator model — {'ISB': 1.01, 'Age': 1.0, 'CTS_months': 1.01}; outcome model — {'CTSE': 1.24, 'ISB': 1.24, 'Age': 1.02, 'CTS_months': 1.01}.

Homoscedasticity (Breusch–Pagan p-values): mediator = 0.00886, outcome = 0.0684.

Residual normality (Shapiro–Wilk p-values): mediator = 0.11, outcome = less than .001.

Influence (Cook’s distance): mediator — max = 0.090, n greater than 4 divided by n = 6; outcome — max = 0.161, n greater than 4 divided by n = 6.

**Figure D1*.*** Mediation effects with 95% confidence intervals


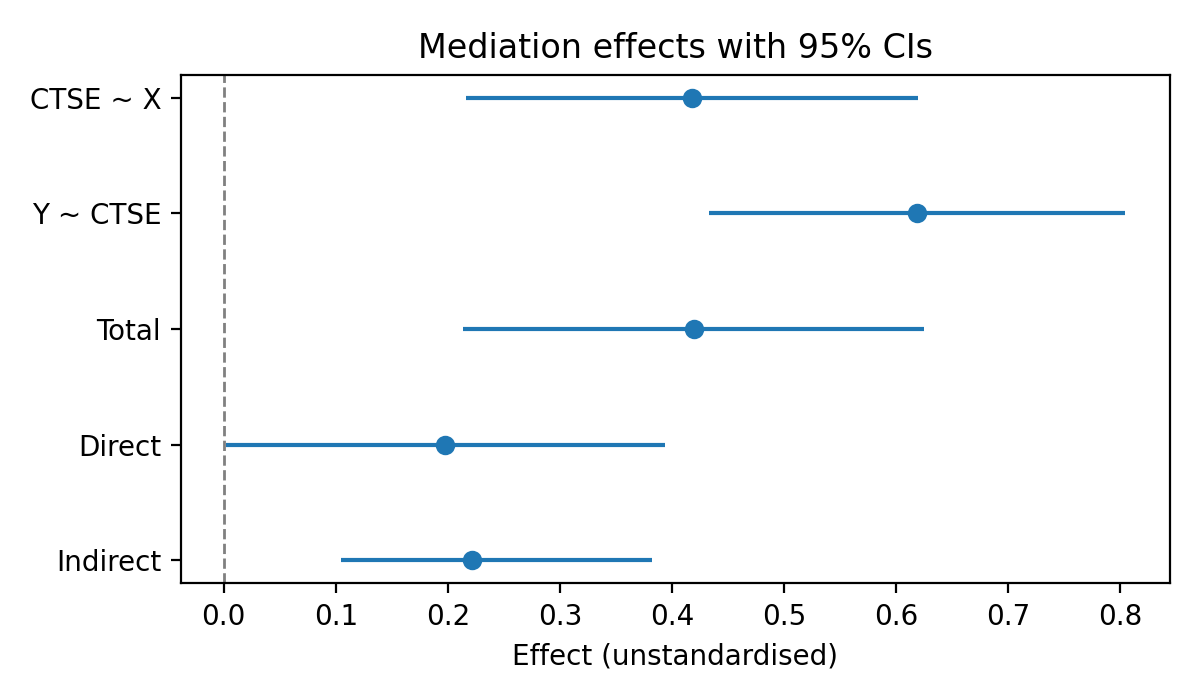

Supplement: Supplementary file 5 [file Datasheet5.docx]
