# Supplementary material for: Contract teaching as a liminal bridge: how pre-entry beliefs become commitment in PE teacher socialisation
Source: Front Sports Act Living. 2025 Dec 18;7:1719826. doi: 10.3389/fspor.2025.1719826 (PMC12756359; doi:10.3389/fspor.2025.1719826)
Supplement: Supplementary file 6 [file Datasheet6.docx]

**Appendix F.**

## Codebook — Q22: What support/preparation would have helped before CTS? (N = 79)

Frequencies are reported as descriptive indicators of salience to aid interpretation; we do not infer prevalence beyond this sample

**Table F1.** Codebook — Q22.

| **Category** | **Brief definition** | **Inclusion rules** | **Exclusion rules** | **Salience**  **n (%)** | **Exemplar extract (ID)** |
| --- | --- | --- | --- | --- | --- |
| Pre‑solo exposure / shadowing | More observation/co‑teaching before taking classes. | 1–2 weeks shadowing; gradual release to solo. | Generic “supportive mentor” (see Q21 Relational). | 8 (10.1) | “More lesson observations… before taking on a class independently.” [TS14] |
| Lesson planning & PE‑specific pedagogy | Worked examples, task progressions, cues. | Templates; exemplars; progression libraries. | Classroom control without planning content (Classroom mgmt). | 7 (8.9) | “Step‑by‑step lesson on how to create a lesson plan would help.” [TS20]; “Examples of lesson plans.” [TS38] |
| Classroom/behaviour management in PE spaces | Routines for large/outdoor classes and transitions. | Signals; equipment flows; safety‑brief scripts. | Assessment/lesson design (Planning). | 4 (5.1) | “Better understanding of classroom management.” [TS02] |
| Orientation depth/length & realism | Clearer preview of school variability and expectations. | What CTS looks like in different schools; role scope. | Generic admin complaints. | 3 (3.8) | “Realistic expectations… CTS is very dependent on school.” [TS77] |
| Inclusion/SEN training | Targeted pre‑CTS SEN/differentiation resources. | Universal design basics; modifying tasks. | General engagement talk. | 1 (1.3) | “More education on special needs.” [TS15] |
| Admin/policies & time management | Navigating paperwork/systems efficiently. | Forms; discipline processes; duty rosters. | Facilities maps (Resources). | 1 (1.3) | “The amount of ‘paperwork’ that had to be done.” [TS55] |
| Facilities/resources orientation | Accessing/booking spaces & equipment; alternatives. | Venue maps; rainy‑day playbook. | Lesson design details. | 1 (1.3) | “Knowing where/what to book would be useful.” [TS11] |
| No additional support needed | Felt adequately prepared. | Explicit “nothing more needed”. | — | 12 (15.2) | “Preparation was sufficient for me.” [TS12] |

Note. Multi‑coding permitted; percentages use N = 79. Quotes anonymised; minimal edits for clarity.
