# Supplementary material for: Contract teaching as a liminal bridge: how pre-entry beliefs become commitment in PE teacher socialisation
Source: Front Sports Act Living. 2025 Dec 18;7:1719826. doi: 10.3389/fspor.2025.1719826 (PMC12756359; doi:10.3389/fspor.2025.1719826)
Supplement: Supplementary file 7 [file Datasheet7.docx]

**Appendix G.**

## Joint display (Quant–Qual integration)

**Table G1.** Joint display JD‑1: Quant–Qual integration of key findings.

| **Quantitative finding** | **Statistical evidence** | **Qualitative mechanism(s)** | **Illustrative extract(s)** | **Integrated inference** |
| --- | --- | --- | --- | --- |
| ISB ↔ CTT correlation | r = .42, p < .01 (N = 79) | Teacher identity & confidence; Student engagement & learning | “Breakthrough with a challenging student…” [TS34]; “Students… love my lesson.” [TS56] | Pre‑entry beliefs gain traction when early wins affirm fit/purpose. |
| ISB ↔ CTS‑E correlation | r = .42, p < .01 | Relational & organisational supports; Pedagogical craft | “Receiving feedback… strengths and areas for growth.” [TS13] | Supportive departments translate inclination into perceived stint quality. |
| CTS‑E ↔ CTT correlation | r = .59, p < .01 | Identity & confidence; Student engagement & learning | “Positive environment strengthened my passion to teach.” [TS33] | High‑quality CTS consolidates commitment via affirming experiences. |
| Mediation (ISB → CTS‑E → CTT) | Partial mediation.  ISB →CTSE → CTT  = 0.22  (95 % CI [0.11, 0.38]); c′ = 0.20, p = .048; *a* = 0.42; *b* = 0.62. | Relational supports; Early wins; Craft learning; buffered by Work realities | “Co‑teaching and feedback from mentors helped me succeed early.” [TS25] | Partial mediation: CTS quality is the hinge turning belief into commitment. |
| Gender difference (CTS‑E) | Male 9.01 (1.07) > Female 8.09 (1.26); t(24.70) = 2.80, p = .010; g = 0.81 [0.28, 1.35] | Work realities; Orientation realism; Management routines | “Having to take a form class… not expected.” [TS76]; “Managing 40 diverse learners.” [TS34] | Differences in role expectations/context may shape perceived stint quality. |

Note. Quant values are mirrored in Tables 2–4; mediation uses bias-corrected bootstrap (5,000) with Age and CTS duration as covariates, with scores on a ten-point scale.
